# What psychometric scales did researchers use to measure dental fear, anxiety, or phobia? A literature survey from 2020 to 2024

**DOI:** 10.3389/froh.2026.1850040

**Published:** 2026-06-11

**Authors:** Deepal Haresh Ajmera, Natalie Sui Miu Wong, Andy Wai Kan Yeung

**Affiliations:** 1 Oral and Maxillofacial Radiology, Applied Oral Sciences and Community Dental Care, Faculty of Dentistry, University of Hong Kong, Hong Kong, Hong Kong SAR, China; 2 College of International Education, Hong Kong Baptist University, Kowloon, Hong Kong SAR, China

**Keywords:** CDAS, CFSS-DS, dental anxiety, dental fear, dental phobia, MDAS, odontophobia

## Abstract

**Background:**

Dental fear, anxiety, and phobia are common psychological constructs that affect patient cooperation and treatment outcomes. Numerous psychometric tools have been developed to measure these constructs, yet contemporary usage patterns remain unclear. This study aimed to survey the psychometric scales used to assess dental fear, anxiety, or phobia in the scientific literature published from 2020 to 2024.

**Methods:**

On 1 October 2025, Web of Science and Scopus were searched using the terms “dental fear,” “dental anxiety,” “dental phobia,” and “odontophobia,” limited to title, abstract, and keywords. Eligible records were English-language original articles published in 2020–2024 that used at least one psychometric tool. After screening and deduplication, 772 papers were included. Each paper was coded for subject age group, psychometric tools used, author departmental affiliation, and publication year.

**Results:**

Nearly half of the studies focused on adults (46.1%), while 45.7% examined children only. Most studies (76.2%) used a single tool. Dentistry-affiliated authors contributed to 93.0% of papers, whereas psychology and psychiatry authors contributed to 10.5% and 3.2%, respectively. The most frequently used tools were the Modified Dental Anxiety Scale (MDAS, 32.3%), Corah's Dental Anxiety Scale (CDAS, 13.7%), and the Children's Fear Survey Schedule–Dental Subscale (CFSS-DS, 10.4%). Comparisons with earlier periods (1988–1998 and 2008) showed a marked rise in MDAS usage and a decline in CDAS usage.

**Conclusions:**

A wide range of psychometric instruments were used in dental fear, anxiety, and phobia research from 2020 to 2024, with MDAS remaining dominant. The limited involvement of psychology and psychiatry researchers suggests opportunities for greater interdisciplinary collaboration to enhance theoretical and measurement rigor.

## Introduction

Dental fear, anxiety, and phobia are prevalent issues that affect individuals across all age groups and have significant implications for oral health outcomes. Dental fear and anxiety can lead to appointment avoidance, irregular attendance, increased treatment complexity, and poorer oral health-related quality of life (1, 2), with an estimated prevalence of 15.3% among adults (3) and 30% among 2–6 year-old children (4). In severe cases, these symptoms may manifest as dental phobia—marked and persistent fear that interferes with daily functioning and reflects the criteria of specific phobia within the mental disorder field. Despite their clinical importance and conceptual differences, the literature seems to use the phrases of dental fear, dental anxiety, and dental phobia interchangeably (especially the former two), with a wide range of psychometric tools being developed to measure them (5, 6).

Over the past several decades, numerous scales have been introduced to quantify dental fear, anxiety, and phobia, including Corah's Dental Anxiety Scale (CDAS) (7), the Modified Dental Anxiety Scale (MDAS) (8), the Dental Fear Survey (DFS) (9), the Children's Fear Survey Schedule–Dental Subscale (CFSS-DS) (10), and several behaviorally oriented measures for pediatric populations [e.g., Frankl Behaviour Rating Scale (11), Venham scales (12)]. These tools vary considerably in format, theoretical grounding, dimensionality, and population specificity. Readers can refer to comprehensive reviews on the availability of psychometric tools to gauge dental fear, anxiety, and phobia (5, 6, 13–15). Earlier literature surveys, most notably Newton and Buck (14) and Armfield (5), highlighted considerable diversity in tool selection as well as emerging shifts in preferred instruments over time (5, 14).

The field has been continuing to evolve, influenced by increasing interest in patient-reported outcomes, cross-cultural adaptations, and updated conceptual models of fear and anxiety. Furthermore, advanced statistical techniques, such as factor analysis and item response theory, have enabled more refined evaluations of scale validity (16–18). Meanwhile, healthcare systems worldwide have placed increasing emphasis on mental health awareness, potentially reshaping the landscape of dental fear, anxiety, and phobia research. However, despite this growth, no comprehensive survey has systematically examined the types of psychometric tools used in the most recent period (2020–2024), a time also shaped by the COVID-19 pandemic and changing dental service patterns.

Several gaps in the literature underscore the need for a contemporary assessment. First, although multiple psychometric tools exist, their actual usage patterns across recent studies are unknown. Some instruments may have become obsolete while newer ones, such as IDAF-4C+ (16), may be gaining traction. Second, earlier reviews did not analyze the disciplinary backgrounds of research teams. As dental fear, anxiety, and phobia are fundamentally psychological constructs, greater involvement from psychology and psychiatry would be expected; yet anecdotal observations suggest that the field remains largely dentistry-driven. Understanding disciplinary contributions could help identify opportunities for interdisciplinary collaboration that may lead to more robust theoretical frameworks and measurement practices. Third, while past surveys examined limited time frames or small sets of journals, the rapid expansion of indexed publications necessitates an updated and more comprehensive analysis.

To address these gaps, this study systematically surveyed all indexed literature published between 2020 and 2024 that used psychometric tools to measure dental fear, anxiety, or phobia. The objectives were:

1. to document the frequency and distribution of psychometric scales used in adult and pediatric studies;
2. to identify trends in tool usage relative to historical benchmarks;
3. to examine the departmental affiliations of contributing authors; and
4. to provide an updated overview of publication patterns across journals.

This contemporary overview will help researchers select appropriate instruments, identify underutilized but theoretically robust tools, and promote greater interdisciplinary engagement to advance the scientific understanding of dental fear, anxiety, and phobia.

## Methods

On 1st October 2025, the Web of Science Core Collection and Scopus electronic literature databases were queried with the following search string: “dental fear” OR “dental anxiety” OR “dental phobia” OR “odontophobia”. Instrument-specific terms (e.g., MDAS, CFSS-DS) were deliberately not included in the search strategy, as such an approach would preferentially capture studies using a limited number of well-known tools while systematically disadvantaging studies employing newer, adapted, or less frequently named instruments. Instead, a construct-based strategy was adopted, followed by full-text screening to identify all psychometric scales used. The search was limited to the title, abstract, and keywords fields of indexed publications. Additional filters were placed to limit the results to documents labelled as articles, written in English, and published during 2020–2024. The two databases yielded 938 and 1,081 articles, respectively. After removing duplicates, 1,322 papers remained. Among the 1,322 papers, 550 of them were excluded after screening due to the following reasons: not original article (*n* = 59), no measure of dental fear, anxiety, or phobia level with a psychometric scale (*n* = 473), not in English (*n* = 2), or no access to the full-text (*n* = 16). Finally, 772 papers entered the analysis.

Then, the 772 papers were manually coded for the following parameters:

1. Subject age: 1 = Adult (>18y); 2 = Pediatric (<18y); 3 = Mixed; 4 = Cannot tell.
2. Psychometric tools used.
3. Any authors from Dentistry department (Yes/No), Psychology department (Yes/No), and Psychiatry department (Yes/No). For simplicity, the latter two were recognized only if the affiliation information has a clear implication. For example, if an author was affiliated with behavioural science unit under Dentistry, the author would be coded as from Dentistry but not Psychology or Psychiatry.
4. Publication year.

Two authors (DA and AY) independently coded the papers. Inter-rater agreement was good (kappa = 0.80). Disagreements were resolved by discussion and mutual consensus.

## Results

The coded data sheet for the 772 papers was provided as Supplementary File 1. Nearly half of the papers (*n* = 356, 46.1%) investigated adults. More than ¾ of the papers (*n* = 588, 76.2%) used a single tool to measure the level of dental fear, anxiety, or phobia. The vast majority (*n* = 718, 93.0%) of the papers involved authors affiliated with Dentistry department. Though there were some fluctuations, the annual publication count was around 100–200 papers. See Table 1 for details of the characteristics of the papers.

**Table 1:** Characteristics of the 772 papers that used psychometric tools to measure the level of dental fear, anxiety, or phobia of participants.

Characteristics	Frequency (% of 772)
*Age group*	
Adults	356 (46.1)
Children	353 (45.7)
Mixed	53 (6.9)
Cannot tell	10 (1.3)
*No. of tools*	
Single tool	588 (76.2)
Multiple tools	184 (23.8)
*Author affiliation*	
With Dentistry	718 (93.0)
With Psychology	81 (10.5)
With Psychiatry	25 (3.2)
*Publication year*	
2020	110 (14.2)
2021	163 (21.1)
2022	149 (19.3)
2023	141 (18.3)
2024	209 (27.1)

**Figure 1:**
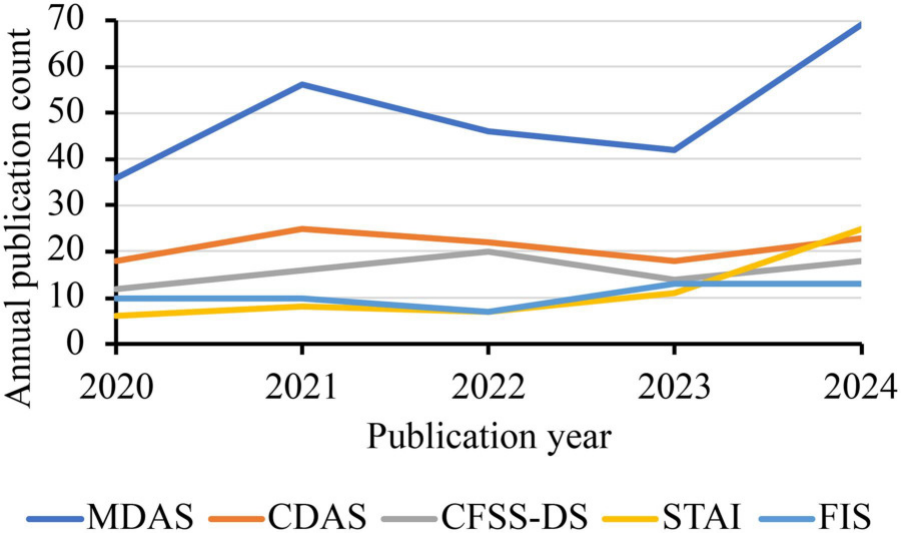
Annual publication counts of the 5 most frequently used psychometric tools to measure the level of dental fear, anxiety, or phobia of participants.

Among all 772 papers, the 5 most frequently used psychometric tools were MDAS (*n* = 249, 32.3%), CDAS (*n* = 106, 13.7%), CFSS-DS (*n* = 80, 10.4%), STAI (*n* = 57, 7.4%), and FIS (*n* = 53, 6.9%). The annual data across this 5-year span showed consistency of their frequent usage (Figure 1). Table 2 lists the top 10 most frequently used psychometric tools used in adult and child studies, respectively. MDAS was the most frequently used tool in adult studies (*n* = 189, 53.1% of 356), whereas CFSS-DS was the most frequently used tool in child studies (*n* = 75, 21.2% of 353). To provide more information, 21 tools that were each used in at least 1% of the analyzed literature set (i.e., at least 8 papers) are listed in Table 3.

**Table 2:** Top 10 most frequently used psychometric tools in adult and child studies.

For adult study	Frequency (% of 356)	For child study	Frequency (% of 353)
MDAS	189 (53.1)	CFSS-DS	75 (21.2)
CDAS	62 (17.4)	FIS	51 (14.4)
STAI	50 (14.0)	VPT	41 (11.6)
DFS	34 (9.6)	MDAS	34 (9.6)
VAS	29 (8.1)	VCAS	30 (8.5)
IDAF-4C+	22 (6.2)	MCDAS	29 (8.2)
Customized	8 (2.2)	MCDASf	28 (7.9)
APAIS	7 (2.0)	CDAS	26 (7.4)
DAI	7 (2.0)	FBRS	24 (6.8)
DAS-R	6 (1.7)	VAS	10 (2.8)

APAIS, Amsterdam Preoperative Anxiety and Information Scale; CDAS, Corah's Dental Anxiety Scale; CFSS-DS, Children's Fear Survey Schedule–Dental Subscale; Customized, customized questionnaire; DAI, Dental Anxiety Inventory; DAS-R, Dental Anxiety Scale Revised; DFS, Dental Fear Survey; FBRS, Frankl Behaviour Rating Scale; FIS, Facial Image Scale; IDAF-4C+, Index of Dental Anxiety and Fear; MCDAS, Modified Child Dental Anxiety Scale; MCDASf, Modified Child Dental Anxiety Scale–Faces; MDAS, Modified Dental Anxiety Scale; STAI, State-Trait Anxiety Inventory; VAS, visual analogue scale; VCAS, Venham Clinical Anxiety Scale; VPT, Venham Picture Test.

**Table 3:** List of psychometric tools to measure dental fear, anxiety, or phobia that were used in at least 1% of the 772 analyzed papers (i.e., at least 8 papers).

Psychometric tool	Frequency (% of 772)
MDAS	249 (32.3)
CDAS	106 (13.7)
CFSS-DS	80 (10.4)
STAI	57 (7.4)
FIS	53 (6.9)
DFS	47 (6.1)
VPT	42 (5.4)
VAS	41 (5.3)
MCDAS	33 (4.3)
VCAS	30 (3.9)
MCDASf	29 (3.8)
IDAF-4C+	27 (3.5)
FBRS	26 (3.4)
Customized questionnaire	14 (1.8)
DAQ	10 (1.3)
DAI	9 (1.2)
RMS-PS	9 (1.2)
Single Likert item	9 (1.2)
VBRS	9 (1.2)
ACDAS	8 (1.0)
SDAI	8 (1.0)

ACDAS, Abeer Children Dental Anxiety Scale; CDAS, Corah's Dental Anxiety Scale; CFSS-DS, Children's Fear Survey Schedule–Dental Subscale; DAI, Dental Anxiety Inventory; DAQ, Dental Anxiety Question; DFS, Dental Fear Survey; FBRS, Frankl Behaviour Rating Scale; FIS, Facial Image Scale; IDAF-4C+, Index of Dental Anxiety and Fear; MCDAS, Modified Child Dental Anxiety Scale; MCDASf, Modified Child Dental Anxiety Scale–Faces; MDAS, Modified Dental Anxiety Scale; RMS-PS, Raghavendra, Madhuri, and Sujata Pictorial Scale; STAI, State-Trait Anxiety Inventory; SDAI, Short Version of Dental Anxiety Inventory; VAS, visual analogue scale; VBRS, Venham Behavior Rating Scale; VCAS, Venham Clinical Anxiety Scale; VPT, Venham Picture Test.

The top 10 journals (including ties) with highest publication count within the analyzed literature set are listed in Table 4. BMC Oral Health was the leading journal (*n* = 44, 5.7%), followed by International Journal of Clinical Pediatric Dentistry (*n* = 29, 3.8%). Most of them were dental journals.

**Table 4:** Top 10 journals (including ties) with highest publication count within the analyzed literature set.

Journal (2024 Journal Impact Factor)	Publication count (% of 772)
BMC Oral Health (3.1)	44 (5.7)
International Journal of Clinical Pediatric Dentistry (N/A)	29 (3.8)
European Archives of Paediatric Dentistry (2.0)	23 (3.0)
Dentistry Journal (3.1)	20 (2.6)
International Journal of Environmental Research and Public Health (N/A)	17 (2.2)
Journal of Indian Society of Pedodontics and Preventive Dentistry (N/A)	17 (2.2)
International Journal of Paediatric Dentistry (1.9)	15 (1.9)
Journal of Clinical Medicine (2.9)	14 (1.8)
Pesquisa Brasileira em Odontopediatria e Clinica Integrada (0.4)	14 (1.8)
European Journal of Oral Sciences (1.8)	12 (1.6)
European Journal of Paediatric Dentistry (2.7)	12 (1.6)
Journal of Oral and Maxillofacial Surgery (2.6)	12 (1.6)
Journal of Pharmacy and Bioallied Sciences (0.9)	12 (1.6)

N/A means the journals were not covered by the 2025 Journal Citation Reports (Clarivate, PA, USA) which lists the 2024 Journal Impact Factor.

## Discussion

This literature survey identified several notable findings in the use of psychometric tools to measure dental fear, anxiety, or phobia between 2020 and 2024. The most prominent finding was the continued dominance of the Modified Dental Anxiety Scale (MDAS), which appeared in nearly one-third of all surveyed papers (32.3%) and was the preferred instrument in adult-focused studies (53.1%). This preference aligns with the long-standing reputation of MDAS for brevity, ease of administration, and strong psychometric characteristics. In contrast, the usage of CDAS has continued its long-term decline. When compared across three periods, 1988–1998 (14), 2008 (5), and 2020–2024 (the current study), the proportion of studies using CDAS fell sharply from 92.1% to 13.7%, whereas MDAS showed the opposite pattern, rising from 2.6% to 32.3% (Table 5). Tools designed for children (e.g., CFSS-DS) and for general anxiety (e.g., STAI) displayed more stable usage patterns.

**Table 5:** Number of papers using various psychometric tools to measure dental fear, anxiety, or phobia in 3 different periods.

Tools	Number of papers (% of *n* from the respective period)		
Period	1988–1998 (*n* = 38)	2008 (*n* = 57)	2020–2024 (*n* = 772)
Data source	Newton and Buck (2000) (14)	Armfield (2010) (5)	This study
MDAS	1 (2.6)	6 (10.5)	249 (32.3)
CDAS	35 (92.1)	19 (33.3)	106 (13.7)
CFSS-DS	2 (5.3)	11 (19.3)	80 (10.4)
STAI	3 (7.9)	5 (8.8)	57 (7.4)
FIS	0	2 (3.5)	53 (6.9)
DFS	4 (10.5)	10 (17.5)	47 (6.1)
VPT	1 (2.6)	1 (1.8)	42 (5.4)
VCAS and VBRS	1 (2.6)	0	VCAS: 30 (3.9) VBRS: 9 (1.2)
FBRS	0	2 (3.5)	26 (3.4)
DAI	4 (10.5)	0	9 (1.2)
SDAI	0	8 (14.0)	8 (1.0)
HAQ	0	1 (1.8)	6 (0.8)
HADS	0	3 (5.3)	3 (0.4)

Tools are listed only if they were mentioned by previous 2 surveys reported in the table, and used in 2 or more papers in at least one period.

CDAS, Corah's Dental Anxiety Scale; CFSS-DS, Children's Fear Survey Schedule–Dental Subscale; DAI, Dental Anxiety Inventory; DFS, Dental Fear Survey; FBRS, Frankl Behaviour Rating Scale; FIS, Facial Image Scale; HADS, Hospital Anxiety and Depression Scale–Anxiety subscale; HAQ, Hierarchical Anxiety Questionnaire; MDAS, Modified Dental Anxiety Scale; SDAI, Short Version of Dental Anxiety Inventory; STAI, State-Trait Anxiety Inventory; VBRS, Venham Behavior Rating Scale; VCAS, Venham Clinical Anxiety Scale; VPT, Venham Picture Test.

Meanwhile, the predominance of single-tool usage may reflect practical considerations such as time constraints, ease of administration, and standardization across clinical settings. However, it may also indicate a degree of methodological simplification, as dental fear, anxiety, and phobia are multidimensional constructs comprising cognitive, behavioural, and physiological components. Reliance on a single instrument, especially unidimensional scales, may therefore limit the ability to capture the full complexity of patient experiences.

An important contextual observation is the disciplinary composition of the authorship. Most studies (93.0%) involved researchers affiliated with Dentistry departments, whereas far fewer involved scholars from Psychology (10.5%) or Psychiatry (3.2%). Although unsurprising given the clinical grounding of dental fear research, this distribution highlights an ongoing disciplinary imbalance. Dental fear, anxiety, and phobia, despite being common causes of treatment avoidance and reduced oral health-related quality of life, have not been explicitly categorized in any edition of the Diagnostic and Statistical Manual of Mental Disorders (DSM-I through DSM-5). Consequently, these conditions are often subsumed by researchers under broader diagnostic entities such as Specific Phobia [e.g., “blood-injection-injury” (19) or “situational” subtypes (20)]. However, this classification may not fully capture the multidimensional nature of the fear or the distinct cognitive, affective, and behavioral profiles observed in dental patients.

Greater involvement from the psychological sciences would likely enrich theoretical understanding in this field, as psychologists bring domain expertise in fear conditioning, avoidance behavior, and the cognitive mechanisms underlying anxiety. Several psychological frameworks may offer valuable explanatory models for dental fear, anxiety, and phobia. Classical conditioning theory suggests that traumatic or painful dental experiences can lead to persistent conditioned anxiety responses (21). Vicarious learning and observational modeling may also contribute when children observe parents or peers reacting fearfully to dental care (22, 23). Cognitive vulnerability models propose that catastrophic thinking, negative appraisal of dental stimuli, and low perceived control are central to dental anxiety (24). More recent studies have also provided relevant psychological constructs, such as sensory sensitivity, pain catastrophizing, and alexithymia (20, 25).

**Figure 2:**
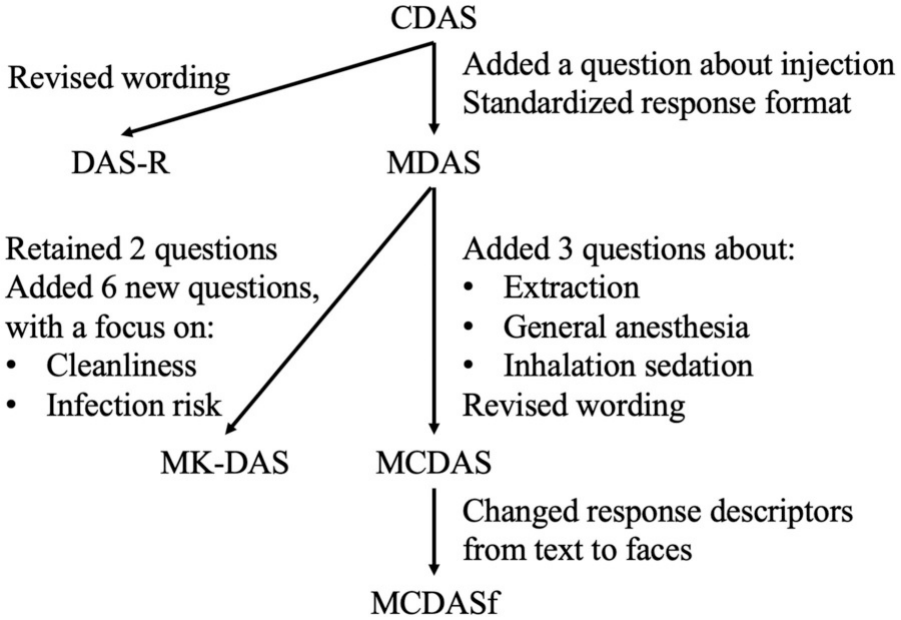
Family of CDAS and its variants identified in the analyzed literature set. CDAS, Corah's Dental Anxiety Scale; DAS-R, Dental Anxiety Scale Revised; MDAS, Modified Dental Anxiety Scale; MK-DAS, Musa Kazim's Dental Anxiety Scale; MCDAS, Modified Child Dental Anxiety Scale; MCDASf, Modified Child Dental Anxiety Scale–Faces.

In contrast, the dental field has traditionally emphasized the application of existing psychometric instruments despite their conceptual limitations, including its primarily unidimensional structure (16). One direction was to revise and create variants of existing tools, such that a family of CDAS and its variants was identified in the selected literature set (Figure 2). More recent measures, such as the Index of Dental Anxiety and Fear (IDAF-4C+), have attempted to incorporate multifactorial conceptual frameworks. The variation in psychometric tools reflects differing conceptualizations of dental fear, anxiety, and phobia. For example, instruments such as the MDAS and CDAS primarily assess situational anxiety related to dental procedures, often capturing anticipatory emotional responses. In contrast, tools such as the IDAF-4C+ aim to incorporate broader domains including emotional, cognitive, behavioural, and physiological aspects. This heterogeneity suggests that different studies may, in effect, be measuring related but non-identical constructs, which may complicate comparisons across the literature. As the mechanisms underlying fear and anxiety reside fundamentally in psychological processes, increased interdisciplinary collaboration with psychology and psychiatry researchers contributing theoretical, experimental, and clinical insights should likely lead to more robust models and more valid assessment tools for dental fear, anxiety, or phobia. Such collaborations can be structured through joint grant initiatives, co-supervision of research postgraduate students, or development of cross-disciplinary special issues in journals.

This study had several limitations. One limitation was that it did not evaluate if the researchers used the named psychometric tools in their original format. Prior literature surveys that focused on a single tool, such as MDAS, Global Quality Score (GQS), and Edinburgh Handedness Inventory (EHI), have found that researchers might change the number/content of the items, response format, cut-off scores and inform the readers about the modifications either implicitly or explicitly (26–29). Regardless, researchers should still try their best to use the tools in their original format, and explicitly tell readers about any modifications. Besides, this literature survey searched through Web of Science and Scopus only. Papers not indexed by these major databases might be omitted. Moreover, some studies might measure physiological signs, such as pulse rate, as proxies of dental fear, anxiety, or phobia level. These studies were excluded. It is true that the current study examined a limited time frame of 5 years only, it still brings value to the literature by examining the COVID-19 pandemic period and the period immediately after, as much more dental anxiety research papers were published over the period of 2020–2024 compared to previous years (30) and captures contemporary tool usage in a rapidly expanding publication landscape. It is also possible that pandemic-related disruptions to dental services and heightened health-related anxiety altered patient perceptions of treatment, potentially influencing the prevalence of dental fear, anxiety, and phobia during this period.

In conclusion, research conducted from 2020 to 2024 showed that adult and pediatric dental fear studies were published in similar numbers and that MDAS remains the most frequently used instrument overall, followed by CDAS, and CFSS-DS. The field continues to utilize a diverse array of psychometric tools, yet most research remains dentistry-driven with limited input from psychology and psychiatry. Strengthening interdisciplinary collaboration and enhancing transparency in scale usage may advance both theoretical understanding and methodological rigor in the measurement of dental fear, anxiety, and phobia.

## Data Availability

The original contributions presented in the study are included in the article/Supplementary Material, further inquiries can be directed to the corresponding author.

## Supplementary material

Supplementary File 1 Coded data sheet for the 772 papers.
